# α-Synuclein is required for sperm exocytosis at a post-fusion stage

**DOI:** 10.3389/fcell.2023.1125988

**Published:** 2023-05-23

**Authors:** Micaela Vanina Buzzatto, María Victoria Berberián, Ary Lautaro Di Bartolo, Diego Masone, Claudia Nora Tomes

**Affiliations:** ^1^ Instituto de Histología y Embriología de Mendoza (IHEM)-CONICET-Universidad Nacional de Cuyo, Mendoza, Argentina; ^2^ Facultad de Ciencias Exactas y Naturales, Universidad Nacional de Cuyo, Mendoza, Argentina; ^3^ Instituto de Ciencias Básicas (ICB)-CONICET-Universidad Nacional de Cuyo, Mendoza, Argentina; ^4^ Facultad de Ingeniería, Universidad Nacional de Cuyo, Mendoza, Argentina

**Keywords:** acrosome exocytosis, cell permeabilization, exocytosis, fusion pore, sperm, α-synuclein

## Abstract

The sperm acrosome is a large dense-core granule whose contents are secreted by regulated exocytosis at fertilization through the opening of numerous fusion pores between the acrosomal and plasma membranes. In other cells, the nascent pore generated when the membrane surrounding a secretory vesicle fuses with the plasma membrane may have different fates. In sperm, pore dilation leads to the vesiculation and release of these membranes, together with the granule contents. α-Synuclein is a small cytosolic protein claimed to exhibit different roles in exocytic pathways in neurons and neuroendocrine cells. Here, we scrutinized its function in human sperm. Western blot revealed the presence of α-synuclein and indirect immunofluorescence its localization to the acrosomal domain of human sperm. Despite its small size, the protein was retained following permeabilization of the plasma membrane with streptolysin O. α-Synuclein was required for acrosomal release, as demonstrated by the inability of an inducer to elicit exocytosis when permeabilized human sperm were loaded with inhibitory antibodies to human α-synuclein. The antibodies halted calcium-induced secretion when introduced after the acrosome docked to the cell membrane. Two functional assays, fluorescence and transmission electron microscopies revealed that the stabilization of open fusion pores was responsible for the secretion blockage. Interestingly, synaptobrevin was insensitive to neurotoxin cleavage at this point, an indication of its engagement in *cis* SNARE complexes. The very existence of such complexes during AE reflects a new paradigm. Recombinant α-synuclein rescued the inhibitory effects of the anti-α-synuclein antibodies and of a chimeric Rab3A-22A protein that also inhibits AE after fusion pore opening. We applied restrained molecular dynamics simulations to compare the energy cost of expanding a nascent fusion pore between two model membranes and found it higher in the absence than in the presence of α-synuclein. Hence, our results suggest that α-synuclein is essential for expanding fusion pores.

## Introduction

Exocytosis is a widespread process that most cells utilize to deliver membranes, lipids, and soluble molecules to the cell surface and the extracellular space. It is mediated by membranous carriers—secretory vesicles—of different sizes and shapes. In regulated exocytosis, vesicles fuse with the plasma membrane in response to extracellular stimuli. This type of exocytosis occurs in specialized cells in order to satisfy specific physiological tasks, such as neurotransmission, respiration, digestion, reproduction, immune responses, and many others (Porat-Shliom et al., 2013). Exocytosis consists of multiple kinetically, functionally and/or morphologically definable stages, the latest of which include docking of secretory vesicles with the plasma membrane and calcium-triggered membrane fusion, followed by release of the granules’ contents (Rizo, 2018; Silva et al., 2021).

During the fusion of a vesicle with the plasma membrane, the lumen of the former initially connects to the extracellular space via a narrow fusion pore (Quevedo et al., 2016; Alvarez de et al., 2018; Karatekin, 2018; Sharma and Lindau, 2018). Low-molecular-weight substances can permeate the narrow pore, which is sometimes stable for several tens of milliseconds before it expands. An Ω-shaped membrane profile may occasionally be captured by electron microscopy. Exocytosis may occur in distinct ways: full-collapse fusion or kiss-and-run. In the former, the fusion pore rapidly dilates, allowing the vesicle membrane to flatten and integrate its lipid and proteins into the planar surface of the plasma membrane. In the kiss-and-run mode, a narrow fusion pore allows the vesicle to retain its gross morphological shape while it releases its contents; the pore is transient and it eventually reseals (Alabi and Tsien, 2013). Large molecules or those packed into dense matrices can only be released by full fusion. Although various members of the fusion machinery have been implicated in fusion pore opening and dilation, the mechanisms involved are far from being elucidated [(Quevedo et al., 2016) and references therein].

The accumulation and aggregation of α-synuclein in the brain characterize devastating neurodegenerative disorders such as Parkinson’s disease, dementia with Lewy bodies, and multiple system atrophy (Masaracchia et al., 2018; Runwal and Edwards, 2021). α-Synuclein is a small (≃15 kDa), cytosolic protein, unstructured in solution, whose amino terminal region folds into two antiparallel α-helices in the presence of highly curved membranes with acidic phospholipid headgroups. Synuclein promotes, as well as senses, membrane curvature (Gallea et al., 2018; Alza et al., 2019). Although the precise normal function of α-synuclein is not well established, evidence toward its involvement in the exocytotic pathway is beginning to emerge [see (Gallea et al., 2018; Alza et al., 2019) for functions attributed to α-synuclein]. Of particular interest to this study is α-synuclein’s involvement in fusion pore dilation, a phenomenon observed in artificial membranes (Khounlo et al., 2021) and during exocytosis in cultured rat hippocampal neurons and mouse adrenal chromaffin cells (Logan et al., 2017).

A discrete number of highly conserved protein families involved in the late stages of the exocytotic cascade has been identified; within them, the SNAREs constitute the core of the fusion machinery in all cells [reviewed in (Barclay et al., 2012; Jahn and Fasshauer, 2012; Rizo and Xu, 2015; Zhang and Hughson, 2021)]. Synaptobrevin-2, syntaxin1, and SNAP-25 families are the synaptic isoforms of the SNARE superfamily. Synaptobrevins are classified as R-SNAREs (arginine-containing SNAREs) and syntaxins and SNAPs as Q-SNAREs (glutamine-containing SNAREs) based on the identity of highly conserved residues (Fasshauer et al., 1998). The Q- and R-SNAREs join into parallel four-helix bundles; Q-SNAREs contribute three helices whereas R-SNAREs contribute the remaining one. When Q-SNARES are located in one membrane and the cognate R-SNARE in the other, complexes are in a *trans* configuration. SNARE proteins zipper progressively from the amino terminus portion of the molecules toward the membranes. The energy barrier for bilayer mixing is overcome by the zippering of *trans* SNARE complexes (Sorensen et al., 2006; Walter et al., 2010; Gao et al., 2012; Rizo, 2022). SNAREs are engaged in a functionally inactive *cis* configuration when all cognates are located on the same membrane. Although they have a role in pre-fusion events (Haynes et al., 1998; Xu et al., 1999a; De Blas et al., 2005; Tomes et al., 2005; Jun and Wickner, 2019), c*is* SNARE complexes are often only viewed as by-products of exocytosis (Sorensen, 2009). Disentangling c*is* complexes so that monomeric SNAREs would be available to engage in productive *trans* complexes requires metabolic energy; this energy is provided by the hydrolysis of ATP catalyzed by NSF. α-SNAP bridges the SNARE complex to NSF and stimulates the ATPase activity of the latter (Ryu et al., 2016; Zhao and Brunger, 2016).

Sperm contain a single, large dense-core secretory granule (the acrosome) whose contents are released by regulated exocytosis (acrosomal exocytosis, AE) in the female tract at fertilization or in response to an increase in calcium levels in the test tube (Vardjan et al., 2013; Chiang et al., 2014). The topology of the AE fits neither full collapse nor kiss and run modes because the acrosomal and sperm’s plasma membranes merge through a unique membrane fusion mechanism. Briefly, the AE proceeds through a series of steps that include the swelling of the acrosome and remodeling of its membrane, the docking of the outer acrosomal to the overlying plasma membrane, and the genesis of pores at the points of apposition (Buffone et al., 2012; Zhao et al., 2016). In most dense-core secretion events, the pores widen and the granule contents discharge. In sperm, however, the granule membrane is as large as the section of the plasma membrane it must fuse with, therefore Ω-shaped structures are never observed. Pore expansion leads to the fenestration of the fusing membranes and joining of pores to generate a reticulum of tubules and hybrid plasma-outer acrosomal membrane vesicles. Exactly at which point after the opening of fusion pores do acrosomal contents begin to be released has been a matter of intense study in the field [reviewed in (Buffone et al., 2014; Foster and Gerton, 2016; Hirohashi, 2016)]. When the vesiculated membranes and acrosomal contents are lost, the inner acrosomal membrane becomes the leading edge of the sperm and the AE is fully accomplished (Harper et al., 2008; Tomes, 2015; Belmonte et al., 2016; La Spina et al., 2016). Although no direct evidence supported the claim, it was believed that sperm fusion pores dilated spontaneously. We have been able to dispute this notion by means of a chimeric protein consisting of the amino-terminal portion of Rab3A fused to the carboxy-terminal portion of Rab22A. Thanks to this tool, we have demonstrated that sperm fusion pores are subjected to post-fusion regulation, as happens in many other cells (Quevedo et al., 2016).

Due to their highly specific, zinc-dependent, proteolytic cleavage of the neuronal isoforms of SNARE proteins, tetanus (TeTx) and botulinum (BoNT) toxins are potent inhibitors of secretory vesicle release (Ahnert-Hilger et al., 2013; Pantano and Montecucco, 2014; Pirazzini et al., 2022). Monomeric SNAREs are sensitive—whereas those in *cis* complexes are resistant—to all toxins (Hayashi et al., 1994). Because SNAREs cycle through toxin-sensitive stages, exocytosis is blocked by neurotoxins in neuroendocrine cells. In contrast, SNAREs do not cycle in capacitated human sperm; instead, they are engaged in toxin-resistant *cis* complexes on both the granule and the cell membranes (De Blas et al., 2005). This is because NSF’s dissociating activity is kept dormant by tyrosine phosphorylation. Once AE is initiated, protein tyrosine phosphatase 1B (PTP1B) dephosphorylates NSF, which derepresses its activity (Zarelli et al., 2009). The monomeric SNAREs thus generated are free to assemble in *trans* and achieve the docking of the acrosome to the plasma membrane (Zanetti and Mayorga, 2009). Monomeric synaptobrevin and that engaged in partially assembled *trans* complexes exhibit differential sensitivity to BoNT/B and TeTx (Xu et al., 1999b; Sorensen et al., 2006; Gaisano, 2014; Bombardier and Munson, 2015). These toxins cleave a peptide bond exposed in both configurations (Schiavo et al., 2000; Fabris et al., 2022; Pirazzini et al., 2022), but to sever the bond toxins must bind their substrate. TeTx binds to the N-terminal, whereas BoNT/B binds the C-terminal portion of synaptobrevin’s coil domain. Because SNARE complex assembly begins at the N-terminus, the TeTx-recognition site is hidden in partially assembled SNARE complexes while the BoNT/B recognition site is exposed. This feature is responsible for synaptobrevin’s differential sensitivity to toxins: TeTx only cleaves free synaptobrevin while BoNT/B also cuts synaptobrevin loosely assembled in complexes (Hua and Charlton, 1999; De Blas et al., 2005; Giraudo et al., 2006; Wu et al., 2016). At a very late stage during the AE, downstream the full zippering of SNARE proteins in *trans* and the subsequent opening of fusion pores, sperm SNAREs are engaged in *cis* complexes (Quevedo et al., 2016).

Here, we combined *in silico* with biochemical, functional and microscopy-based methods employing recombinant proteins, neurotoxins, lectins and antibodies to show that human sperm α-synuclein is required for fusion pore expansion during a very late stage of the AE.

## Materials and methods

### Reagents

Recombinant streptolysin O (SLO) was obtained from Dr. Bhakdi (University of Mainz, Mainz, Germany). The rabbit polyclonal antibody directed towards human α-synuclein (purified on protein A) was from Axxora, LLC (Farmingdale, NY). The mouse monoclonal anti-synaptobrevin-2 antibody (clone 69.1, purified IgG) and the rabbit polyclonal anti-complexin I/II (purified IgG) were from Synaptic Systems (Göttingen, Germany). HisTrap columns, FF, Cytiva (formerly GE healthcare Life Sciences) were purchased from ALLSCIENCE, LLC (Doral, FL). FITC-coupled *Pisum sativum* agglutinin (FITC-PSA) was from Vector Labs (BIOARS S.A, Buenos Aires, Argentina). PSA coupled to 20 nm colloidal gold was from glycoMatrix (Dublin, OH). Horseradish peroxidase- and Cy™3-conjugated goat anti-rabbit, as well as Cy™3-conjugated goat anti-mouse IgGs (H + L) were from Jackson ImmunoResearch (West Grove, PA). *O*-nitrophenyl EGTA-acetoxymethyl ester (NP-EGTA-AM) was purchased from Life Technologies (Buenos Aires, Argentina). Ni-NTA-agarose was from GE Healthcare. Prestained molecular weight markers were from Bio-Rad (Tecnolab, Buenos Aires, Argentina). All other chemicals were from Sigma-Aldrich™ Argentina S.A., Genbiotech, or Tecnolab (Buenos Aires, Argentina).

### Recombinant proteins

The light chains of BoNT/B and TeTx fused to His_6_ in a pQE3 vector were generously provided by Dr. T. Binz (Medizinische Hochschule Hannover, Hannover, Germany). The cDNA encoding human α-synuclein fused to His_6_ and to the TAT-transduction domain of HIV virus in a pET11a plasmid was a kind gift of Dr. L. Pollegioni (Università degli Studi dell’Insubria, Varese, Italy). Purified, recombinant Rab3A-22A fused to glutathione S-transferase was a kind gift from Dr. M.F. Quevedo (IHEM, Mendoza, Argentina).

Expression and purification of recombinant His_6_-BoNT/B and His_6_-TeTx were as in (De Blas et al., 2005). The plasmid encoding His_6_-TAT-α-synuclein was transformed into *Escherichia coli* BL21 (DE3) and protein expression was induced with 0.5 mM IPTG 4 h at 37°C. Recombinant-α-synuclein is mostly located in the periplasmic space (Ren et al., 2007). We recovered the periplasm of transformed bacteria by osmotic shock as in (Caldinelli et al., 2013), made it 20 mM TrisHCl, pH 7.4 and run it through a metal-chelating HisTrap column. Twenty volumes of 20 mM TrisHCl, pH 7.4, 1 M NaCl, and 5 volumes each of 20 mM TrisHCl, pH 7.4 plus 125 mM, 250 mM and 500 mM imidazole were applied to the column. Purified His_6_-TAT-α-synuclein eluted with 500 mM imidazole was added 10% glycerol, aliquoted and stored at −80°C until use.

### Human sperm sample preparation procedures

Semen samples were donated by normal healthy men. Semen was allowed to liquefy by incubating at 37°C for 30–60 min. Highly motile cells were isolated from semen following a swim-up protocol. Sperm concentrations were adjusted to 7 × 10^6^/mL before incubating for at least 2 h under capacitating conditions (37°C, 5% CO_2_/95% air) in Human Tubal Fluid media (Serendipia Lab, Vedia, Argentina) supplemented with 0.5% bovine serum albumin (BSA, HTF media). Sperm were permeabilized with SLO as follows: after washing twice with PBS, sperm were incubated for 15 min at 4°C in cold PBS containing 3 U/mL SLO. Cells were washed once with PBS, resuspended in ice-cold sucrose buffer (20 mM Hepes-K, pH 7, 250 mM sucrose, 0.5 mM EGTA) and permeabilized by activating the SLO bound to their plasma membrane with 2 mM DTT. The reagents to test were added sequentially as indicated in the figure legends, and incubated for 8–15 min at 37°C after each addition. Samples were processed for AE assays, immunofluorescence and for transmission electron microscopy. To obtain the results shown in Figure 2B, photosensitive NP-EGTA-AM was supplied to SLO-permeabilized sperm at the beginning of the experiments, ensuring that all subsequent procedures were carried out in the dark. After the last incubation, photolysis was induced by exposing twice (1 min each) to an UV lamp, mixing, and incubating for 5 min at 37°C.

### Indirect AE assay

**FIGURE 1:**
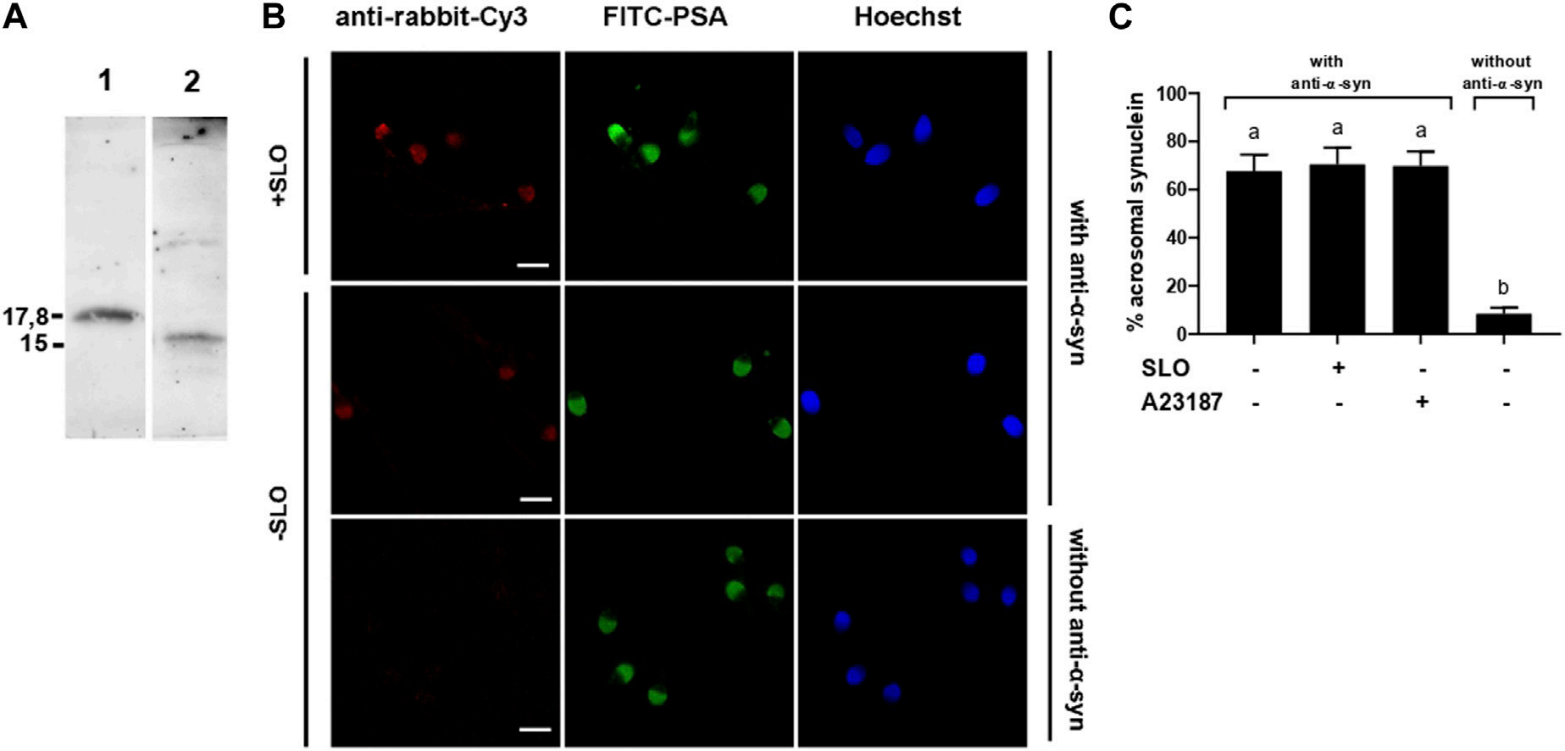
α-synuclein is present in the acrosomal domain of human sperm that retain the acrosome. **(A)** 0.7 µg of recombinant, purified α-synuclein (lane 1) and proteins from unfractionated sperm extracts (20 × 10^6^ cells, lane 2) were immunoblotted with anti-α-synuclein antibodies. Shown are Western blots representative of three repetitions. **(B)** SLO permeabilized (+SLO) and non permeabilized (-SLO) capacitated human sperm were triple stained with (top and middle rows) or without (bottom row, negative control) antibodies to α-synuclein followed by a fluorescent secondary antibody (anti-rabbit-Cy3, red, left panels), FITC-PSA (to assess the integrity of the acrosome; green, central panels), and Hoechst 33,342 (to visualize all cells in the field; blue, right panels). Shown are epifluorescence micrographs of typically stained cells. Bars = 5 μm. **(C)** Quantification of the percentage of non-exocytosed sperm with α-synuclein staining. + SLO: SLO-permeabilized sperm; + A23187: non-SLO-permeabilized cells challenged with a calcium ionophore, an AE inducer. The data represent the mean ± SEM of at least three independent experiments. Statistical difference between the groups was non-significant (*p* > 0.05) except when comparing with the background control without primary antibodies.

Sperm were spotted on teflon-printed slides, air dried, and fixed/permeabilized in ice-cold methanol for 20 s. Acrosomal status was assessed by staining with 25 μg/mL FITC-PSA in PBS for 40 min at room temperature and washing for 20 min in water (Mendoza et al., 1992). PSA binds α-methyl mannoside moieties in proteins present in the acrosomal matrix (Cross et al., 1986). At least 200 cells per condition were scored manually using an upright Zeiss microscope equipped with epifluorescence optics. All experiments included a basal (no stimulation) and a positive (0.5 mM CaCl_2_ [corresponding to 10 μM free calcium]) controls. AE indices were calculated by subtracting the number of spermatozoa that lost their acrosome spontaneously (“control”; assigned 0% for normalization) from all values and expressing the results as a percentage of the AE observed with 0.5 mM CaCl_2_ (“Ca”, assigned 100% for normalization). Our analysis only included results derived from experiments that produced similar responses and where the difference between basal (7%–19% before normalization) and stimulated conditions was of at least 8–10 percentage points (Supplementary Figure S1B). SLO did not affect the PSA staining patterns (see images of PSA-stained SLO-permeabilized sperm in Supplementary Figure S1A; compare “-SLO and “+SLO” FITC-PSA panels in Figure 1B). Data were evaluated before normalization with the program GraphPad Prism 8, using the one way Anova, Dunnett’s multiple comparisons test. Bar plots represent the mean ± SEM of at least three independent experiments. Different letters indicate statistical significance (*p* < 0.05).

### Direct AE assay

SLO-permeabilized sperm were bathed in 30 μg/mL FITC-PSA for all the incubation time. Cells were loaded with 5 nM anti-α-synuclein antibodies followed by 0.5 mM CaCl_2_ and incubated for 15 min at 37°C after each addition. At the end of the second incubation, sperm were spotted on teflon-printed slides, air dried overnight and fixed/permeabilized in 2% paraformaldehyde for 10 min, followed by a 5 min-wash in water. AE indices and statistical analysis were as described above.

### Indirect immunofluorescence

For anti-synaptobrevin-2 immunofluorescence, capacitated and SLO-permeabilized sperm samples were processed as described for indirect AE assays, adding reagents and CaCl_2_ sequentially, and incubating for 10 min at 37°C after each addition. α-Synuclein immunofluorescence was performed on cells subjected or not to SLO permeabilization. Samples were fixed in 2% paraformaldehyde in PBS for 15 min at room temperature, centrifuged and resuspended in PBS containing 100 mM glycine to neutralize the fixative. Cells (5 × 10^5^ per condition) were attached to poly-L-lysine-coated, 12 mm round coverslips by incubating for 30 min at room temperature in a moisturized chamber. Exposure to 0.1% Triton X-100 in PBS for 10 min at room temperature was used to permeabilize the plasma membrane. After the detergent, sperm were washed three times with PBS containing 0.1% polyvinylpyrrolidone (PVP, average M.W. = 40,000; PBS/PVP). Incubation in 3% BSA in PBS/PVP for 1 h at 37°C was used to reduce nonspecific staining. Anti-α-synuclein (10 μg/mL) and anti-synaptobrevin-2 (25 μg/mL) antibodies were diluted in 2% BSA in PBS/PVP, added to the coverslips, and incubated for 1 h at 37°C in a moisturized chamber. After washing (three times, 6 min each) with PBS/PVP, 2.5 μg/mL Cy™3-conjugated anti-rabbit (synuclein) or mouse (synaptobrevin) IgGs in 1% BSA in PBS/PVP) were added and incubated for 1 h at room temperature protected from light. Coverslips were washed (three times, 6 min each) with PBS/PVP. The acrosomal membrane was permeabilized with ice-cold methanol for 1 min, and the acrosomal contents stained with FITC-PSA as described in “Indirect AE assay” (but without air drying). Coverslips were washed (three times, 10 min each) with PBS/PVP, mounted with Mowiol^®^ 4–88 in PBS containing 2 μM Hoechst 33342, and stored at room temperature in the dark. Samples were examined with an 80i Nikon microscope equipped with a Plan Apo 60x/1.40 oil objective. Images were captured with a Nikon DS-Fi1 camera operated with NIS software (Nikon). ImageJ (freeware from N.I.H.) was used to subtract background and adjust brightness/contrast to render all-or nothing labeling patterns. The presence of immunostaining in the acrosomal region was scored in digital images from at least ≈100 cells contained in 10 fields. Data were evaluated before normalization with the program GraphPad Prism 8 using the one way Anova, Dunnett’s multiple comparisons test.

### SDS-PAGE and western blot

Proteins were resolved by electrophoresis on 15% Tris-glycine-SDS-gels and electro-transferred to 0.22 μm nitrocellulose membranes (Hybond, GE Healthcare) on a semi-dry apparatus (Amersham Biosciences) for 35 min at 25 mA. Because α-synuclein monomers tend to detach from blotted membranes, resulting in no or very poor detection (Lee and Kamitani, 2011), membranes were treated with 0.4% paraformaldehyde for 30 min at room temperature immediately after transfer. Non-specific reactivity was blocked with 2% BSA dissolved in washing buffer (PBS, pH 7.6, 0.2% Tween 20) for 1 h at room temperature. Blots were incubated with 1 μg/mL anti-α-synuclein in blocking solution overnight at 4°C. Horseradish peroxidase-conjugated goat-anti-rabbit IgG (0.1 μg/mL in washing buffer) was used as secondary antibody with 1 h incubation at room temperature. Excess first and second antibodies were removed by rocking three times, 10 min each in washing buffer. Detection was accomplished with a chemiluminescence kit from Kalium Technologies (Biolumina, Buenos Aires, Argentina) on a Luminescent Image Analyzer LAS-4000 (Fujifilm, Tokyo, Japan).

### Transmission electron microscopy

For experiments summarized in Figure 4, we processed capacitated and SLO-permeabilized human sperm (5 × 10^6^ cells in 250 µL per condition) as described for “Indirect AE assays”. For experiments summarized in Figure 5, we incubated the cells with 20 μg/mL PSA-colloidal gold as described in the legend. Reactions were stopped by fixing in 2.5% glutaraldehyde in 0.1 M sodium phosphate buffer and incubating overnight at 4°C or 2 h at room temperature. Samples were washed in PBS (three times, 20 min each) and postfixed in 1% OsO_4_ in PBS for 1 h at room temperature, washed as before, dehydrated in a graded acetone series and embedded in a low viscosity epoxy resin (Pelco International, Fresno, CA) (Spurr, 1969). Polymerization was performed at 70°C for 48 h. Ultrathin sections with interference color gray were cut with an ultramicrotome (Ultracut R; Leica, Wien, Austria), mounted on grids and stained with uranyl acetate and lead citrate (Reynolds, 1963). Grids were examined by transmission electron microscopy at 80 kV in a 900 Zeiss microscope (Jena, Germany). We included negative (not stimulated) and positive (stimulated with 0.5 mM CaCl_2_) controls in all experiments.

### Computational methods

We conducted umbrella sampling molecular dynamics using GROMACS (GROningen Machine for Chemical Simulations)-2020.5 (Van Der et al., 2005; Pronk et al., 2013), PLUMED-2.7.2 (Tribello et al., 2014) and the Martini 3 coarse-grained model (Souza et al., 2021). In all cases, we modeled two 1024-lipid bilayers patches composed of 87.5% of 1-palmitoyl-2-oleoyl-glycero-3-phosphocholine (POPC), 10% of 1-palmitoyl-2-oleoyl-sn-glycero-3-phospho-L-serine (POPS) and 2.5% of the recently developed phosphatidylinositol 4,5-bisphosphate (PI(4,5)P_2_, or PIP_2_) model (Borges-Araujo et al., 2022). This arrangement, previously used in our laboratory (Di Bartolo et al., 2022; Di Bartolo and Masone, 2022) follows the experimental membrane composition reported by Jahn and collaborators to trap Syt1 to the plasma membrane in the presence of calcium (Perez-Lara et al., 2016). These bilayer patches of ∼17 × 17 nm ensure negligible finite-size effects due to interactions between periodic images of the fusion pore (Caparotta et al., 2020b). Solvation fulfilled the ample water condition for Martini (Ingolfsson et al., 2014) and the inter-membrane separation was adjusted to equilibrate at ∼3.9 nm (Di Bartolo and Masone, 2022). See Supplementary Material for more technical details.

## Results

### α-Synuclein is localized to the acrosomal region of human sperm

We investigated the presence of α-synuclein in human sperm by Western blot using a polyclonal antibody raised against recombinant human α-synuclein as probe. Immunoblot analysis of whole cell extracts detected a single protein band with an apparent molecular mass of 15 kDa (Figure 1A, lane 2). The recombinant protein used as positive control showed decreased electrophoretic mobility compared to that of the endogenous form (Figure 1A, lane 1). This is because α-synuclein expressed in *E. coli* contains the TAT peptide and therefore has a higher molecular weight (≃ 17.8 kDa) than sperm α-synuclein.

To explore the localization of α-synuclein in sperm, we performed indirect immunofluorescence experiments using anti-α-synuclein as primary and anti-rabbit-Cy3 IgGs as secondary antibodies (Figure 1B, red, left panels). FITC-coupled PSA, a lectin that binds the acrosomal matrix, allowed visualization of the acrosomes (Figure 1B, green, central panels). Hoechst 33342 stained the nuclei and accounted for all cells in each field (Figure 1B, blue, right panels). α-Synuclein was detected in the acrosomal region of the sperm head in over 60% of the cells (Figure 1C); this is the expected localization for a protein with a role in exocytosis. No head staining was observed in sperm that lost their acrosomes either spontaneously (6%) or upon inducing AE with the calcium ionophore A23187 (20%) (not shown). These results suggest that the subcellular compartments that bear α-synuclein shed in cells that undergo exocytosis. Very few cells unexposed to primary antibodies showed fluorescence in the acrosomal domain, indicating that the staining was specific (Figures 1B, C). The percentage of cells labeled by the anti-α-synuclein antibodies was identical whether or not the plasma membrane had been permeabilized with SLO, which indicated that despite its small size, α-synuclein did not diffuse out of cells upon treatment with SLO (Figure 1C).

### α-Synuclein is required for the human sperm AE at a late stage

**FIGURE 2:**
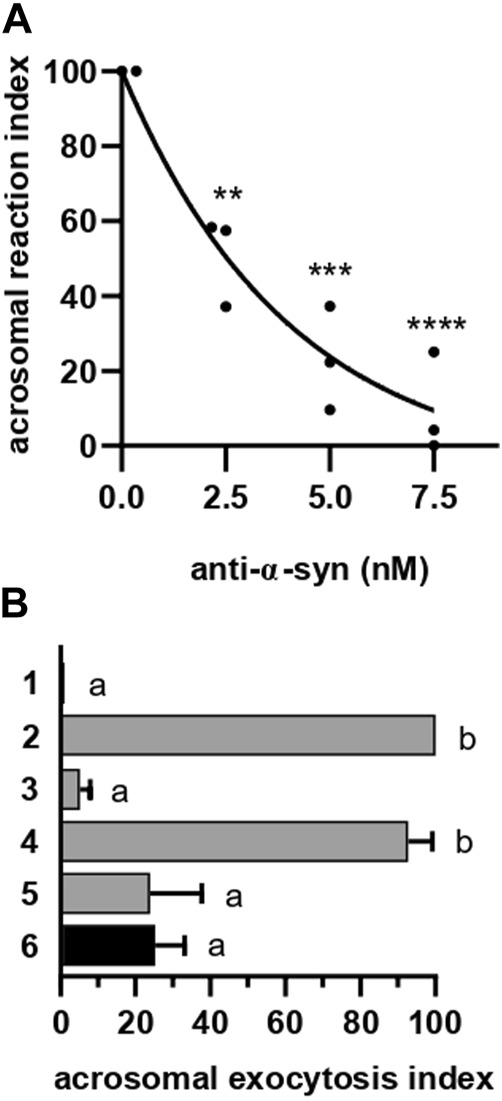
α-synuclein is required for the AE at a late stage. **(A)** Anti-α-synuclein antibodies were introduced into SLO-permeabilized sperm incubating at 37°C for 15 min before initiating the AE by adding 0.5 mM CaCl_2_ and incubating as before. After fixing, the AE was measured as described in “indirect AE assay”. Data were evaluated before normalization with the program GraphPad Prism 8 using the one way Anova, Tukey’s test multiple comparisons test. Statistically significant differences against the control without anti-α-synuclein antibodies were **p < 0.02, ***p < 0.0002 and ****p < 0.0001. **(B)** Permeabilized spermatozoa were loaded with 10 μM NP-EGTA-AM (NP) for 10 min at 37°C before initiating the AE with 0.5 mM CaCl_2_. After another incubation at 37°C, sperm were treated with 5 nM anti-α-synuclein antibodies. All procedures were carried out protecting the tubes from the light. Photolysis of the chelator by UV light (*hv*) was induced at the end (6: NP→Ca^2+^→ anti-α-syn→*hv*, black bar). Gray bars represent controls: 1 = background; 2 = AE stimulated by 0.5 mM CaCl_2_; 3 = inhibitory effect of NP in the dark (NP→Ca→dark); 4 = the recovery upon illumination (NP→Ca→*hv*); 5 = inhibitory effect of the antibodies when present throughout the experiment (NP→ anti-α-syn→Ca→*hv*). The AE was measured as in “**A**”.

To investigate the requirement of α-synuclein for AE, we introduced anti-α-synuclein antibodies into SLO-permeabilized sperm before inducing exocytosis with calcium. The antibodies inhibited the AE in a dose-response fashion, reaching 77% inhibition at 5 nM and 90% inhibition at 7.5 nM (Figure 2A). Preincubation of the antibody with recombinant α-synuclein abolished the IgG’s ability to prevent the AE (Supplementary Figure S2). His_6_-TAT-α-synuclein did not affect either the spontaneous or calcium-induced AE. Nor did imidazole, used in the purification of the protein, or a nonimmune rabbit IgG (Supplementary Figure S2). Antibodies to complexin bind the endogenous protein and decorate the acrosomal domain of the head in human sperm (Roggero et al., 2007). In contrast to anti-α-synuclein antibodies, 7.5 nM anti-complexin (cpx) antibodies did not affect the AE (Supplementary Figure S2). Thus, we concluded that the negative effect of the anti-α-synuclein antibodies on sperm secretion was due to binding to endogenous α-synuclein and therefore specific. Our results revealed that α-synuclein exhibits a role in human sperm AE.

The acrosome behaves as an internal calcium reservoir; release from this store is necessary for the AE [reviewed in (Darszon et al., 2011; Correia et al., 2015)] after the outer acrosomal membrane docks to the plasma membrane and before they fuse (De Blas et al., 2005; Zanetti and Mayorga, 2009; Rodriguez et al., 2011). NP-EGTA-AM is a membrane permeant, photolabile calcium chelator that crosses the plasma (because it is permeabilized with SLO) and outer acrosomal (due to the AM moiety) membranes. After this, the AM group is removed and NP-EGTA accumulates inside the acrosome, where it halts the AE by chelating intra-acrosomal calcium for as long as the system is kept in the dark. Photolysis of the chelator by exposure to UV light rapidly replenishes the acrosomal calcium pool and allows exocytosis to resume (De Blas et al., 2002; Ackermann et al., 2008; Hu et al., 2010; Sosa et al., 2015). In sperm loaded with NP-EGTA-AM and kept in the dark, challenging with CaCl_2_ allowed exocytosis to advance up to the stage that requires intracellular calcium mobilization. Subsequent incubation with anti-α-synuclein antibodies prevented exocytosis even after illuminating the tubes to photolyse the chelator (Figure 2B, black bar). These results indicate that α-synuclein is required at a late stage of the AR, likely after docking and intra-acrosomal calcium release. Interestingly, this is the same timeframe in which the chimeric protein Rab3A-22A halts the AE (Bustos et al., 2014; Quevedo et al., 2016).

### Sperm SNAREs are engaged in *cis* complexes by the time α-synuclein exhibits its role in the AE

**FIGURE 3:**
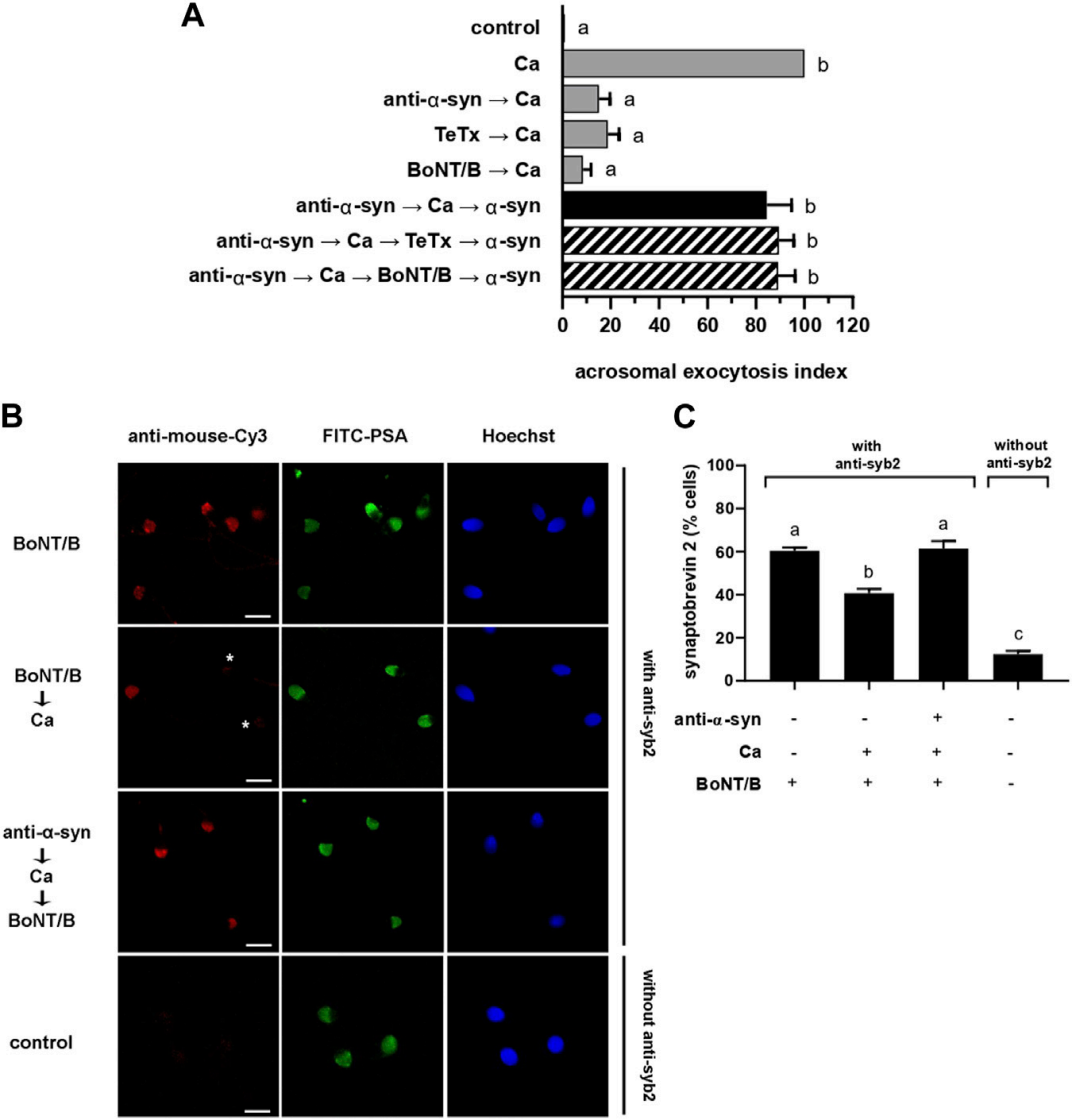
anti-α-synuclein antibodies halt exocytosis at a stage when synaptobrevin-2 is engaged in *cis* SNARE complexes. **(A)** Sperm with their plasma membrane permeabilized with SLO were incubated sequentially with 7.5 nM anti-α-synuclein antibodies, 0.5 mM CaCl_2_, and 20 nM recombinant α-synuclein (15 min at 37°C after each addition; black bar). When indicated, 100 nM TeTx or BoNT/B were added after CaCl_2_ and before recombinant α-synuclein (striped bars). Gray bars represent controls. The AE was measured as indicated in the legend to Figure 2A. **(B)** Sperm incubated as indicated in the figure key, were fixed and triple stained without (bottom row, negative control) or with (all the rest) an anti-synaptobrevin-2 (syb2) antibody followed by a fluorescent secondary antibody (anti-mouse-Cy3, red, left panels), FITC-PSA (green, central panels), and Hoechst 33,342 (blue, right panels). Shown are representative images of sperm with intact acrosomes i) with synaptobrevin-2 staining or ii) without synaptobrevin-2 immunostaining due to toxin cleavage (asterisks). Bars = 5 μm. **(C)** Quantification of the percentage of non-exocytosed sperm with synaptobrevin-2 staining. The left to right order of the bars corresponds to the top to bottom order of the pictures in panel **(B)**. The data represent the mean ± SEM of at least three independent experiments. Statistical difference between the groups was non-significant (*p* > 0.05) except when comparing the % sperm with intact synaptobrevin between the condition BoNT/B →Ca against all the rest and when comparing synaptobrevin-2 labeling against the background control without primary antibodies (*p* < 0.05).

When introduced into SLO-permeabilized human sperm, recombinant α-synuclein reversed the exocytotic block imposed by the anti-α-synuclein antibodies on the AE (Figure 3A, black bar). The reversibility of the effect of the antibodies by bacterially expressed α-synuclein was instrumental to interpret the findings described below.

Results summarized in Figure 2B indicated that α-synuclein controls a post-docking stage during the human sperm AR. Molecularly, docking corresponds to a state where the outer acrosomal and plasma membranes are bridged together by partially assembled *trans* SNARE complexes in preparation for fusion. When in this configuration, SNARE proteins are sensitive to BoNTs but resistant to TeTx. To scrutinize the configuration of the SNARE complex at the post-docking stage governed by α-synuclein, we combined anti-α-synuclein antibodies, BoNT/B and TeTx in a functional assay (Figure 3A). When reagents were introduced into SLO-permeabilized sperm in the sequence anti-α-synuclein → calcium → BoNT/B or TeTx → α-synuclein, the AE proceeded normally (Figure 3A, striped bars). These results revealed that synaptobrevin-2 was protected from either toxin cleavage, which only happens when engaged in SNARE complexes in a *cis* configuration.

We have established in the past that monitoring SNARE proteins’ sensitivity to neurotoxins by indirect immunofluorescence is a suitable assay to determine their configuration. Following a triple staining strategy similar to that used to gather the data compiled in Figures 1B, C, we scrutinized if the introduction of anti-α-synuclein antibodies into SLO-permeabilized sperm affected the susceptibility of synaptobrevin-2 to BoNT/B. The monoclonal antibody used in these experiments recognizes an epitope located in a portion of the molecule severed by the toxin and therefore detects intact—but not BoNT/B-cleaved—synaptobrevin-2. Representative images are shown in Figure 3B. As we have reported before, the anti-synaptobrevin-2 antibody decorated the acrosomal region despite pretreatment with BoNT/B because SNAREs are in a *cis* configuration in resting sperm. When sperm were treated with the toxin plus CaCl_2_, the proportion of cells exhibiting synaptobrevin-2 labeling dropped significantly because the initiation of the AE sensitized this SNARE to BoNT/B. In contrast, when anti-α-synuclein antibodies were introduced into sperm before CaCl_2_, synaptobrevin was insensitive to BoNT/B cleavage (Figure 3C). These results suggest that when CaCl_2_ advanced the AE until it reached the stage blocked by the anti-α-synuclein antibodies, synaptobrevin was in a BoNT/B-resistant configuration. Once again, our findings indicate that the anti-α-synuclein antibodies halted the AE at a stage when SNAREs were likely engaged in a *cis* configuration. This arrangement is characteristic of two exocytotic stages: an early one in resting cells and a late, post-fusion, one. Only the latter is consistent with the findings reported here and therefore we conclude that α-synuclein exhibits its role in the AE after the opening of fusion pores between the acrosomal and plasma membranes.

### α-Synuclein exhibits its role in the AE after the opening of fusion pores

**FIGURE 4:**
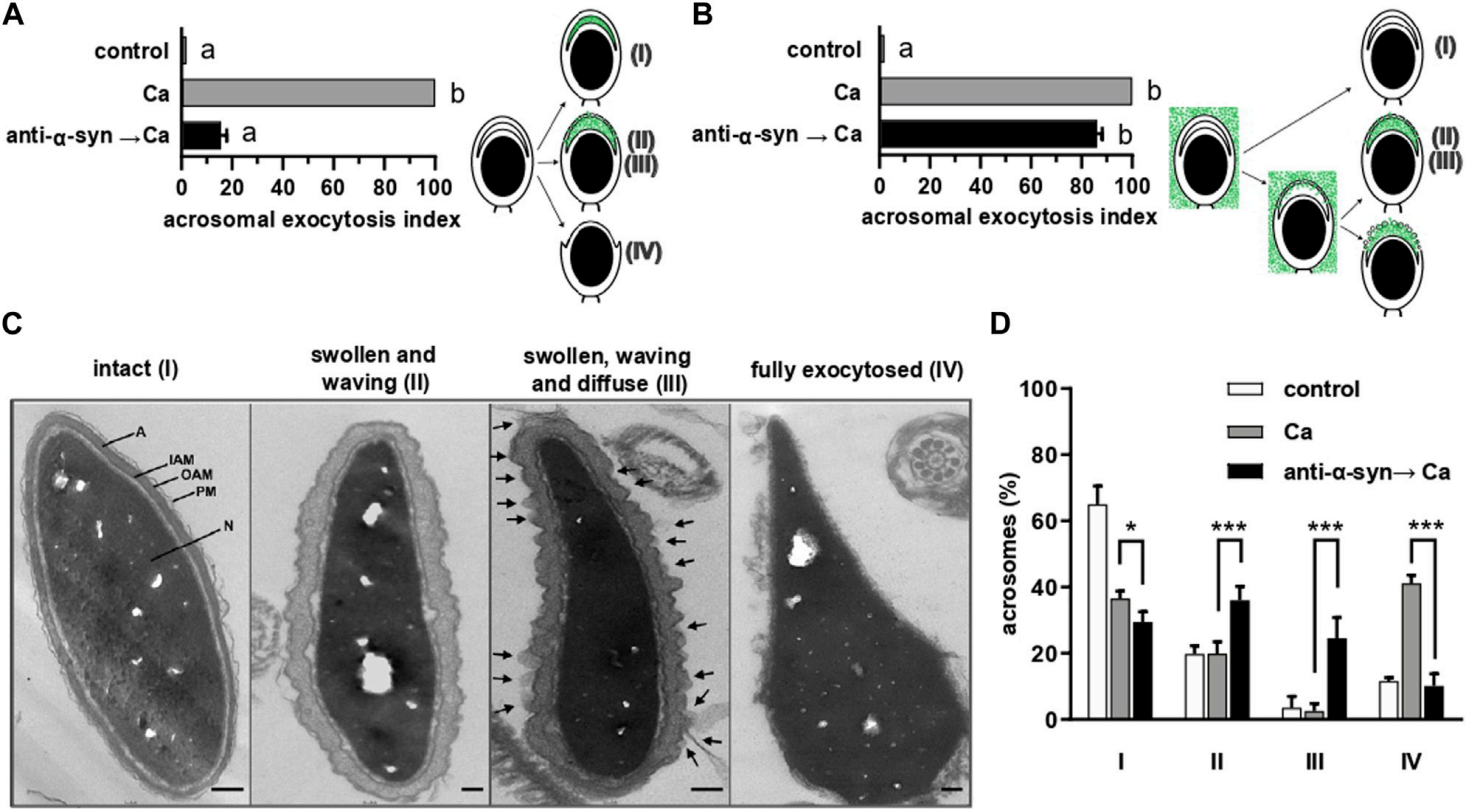
treatment with anti-α-synuclein antibodies allows the efflux of electron dense material from the acrosome of sperm undergoing AE through stabilized fusion pores. **(A)** Sperm were treated with 5 nM anti-α-synuclein antibodies and 0.5 mM CaCl_2_ and incubated 15 min at 37°C after each addition. After fixing, the AE was measured as described in the legend to Figure 2A. The diagram represents the staining patterns of sperm with intact acrosomes, and undergoing intermediate exocytosis stages and after losing the acrosome. Roman numerals are as in panel **(C)**. **(B)** Spermatozoa were bathed in 30 μg/mL FITC-PSA and incubated (15 min at 37°C after each addition) with 5 nM anti-α-synuclein antibodies and 0.5 mM CaCl_2_. The AE was measured as described in “direct AE assay”. The diagram represents the staining patterns of sperm with intact acrosomes, and undergoing intermediate exocytosis stages. Roman numerals are as in panel **(C)**. With this staining method, exocytosis halts at the vesiculated stage, more advanced than II and III but never reaching stage IV. **(C)** Transmission electron micrographs of human sperm heads representative of the four patterns distinguished in this study. Sperm with a flat acrosome (intact, I), sperm with swollen and waving acrosome (II), sperm with swollen and waving acrosome plus electron dense material diffusing out of the cell (III, arrows point to this material), sperm that lost its acrosome completely (fully exocytosed IV). Bars = 200 nm. A acrosome; IAM: inner acrosomal membrane; OAM: outer acrosomal membrane; PM: plasma membrane; N: nucleus. **(D)** Frequency histogram of the percentage of sperm in each of the four categories shown in **C** when cells were incubated with 0.5 mM CaCl_2_ (Ca, gray bars) or pretreated with 15 nM anti-α-synuclein antibodies before adding 0.5 mM CaCl_2_ (anti-α-syn→Ca, black bars). Open bars (control) represent the percentage of sperm in each category in untreated samples. The data represent mean values ±SEM, calculated from four independent experiments with at least 100 cells analyzed in each repetition. Two-way ANOVA shows statistically significant differences between “anti-α-syn→Ca” vs. “Ca” groups for all the samples analyzed, *p < 0.05 and ***p < 0.0001 (Tukey’s test).

Anti-α-synuclein antibodies froze sperm SNAREs in toxin-resistant, *cis* complexes (Figure 3). We used fluorescence and transmission electron microscopies to further explore the premise that the antibodies affect a post-fusion event. FITC-PSA reveals the presence of the acrosomal matrix *after fixation* in the standard (indirect) functional assay: sperm without acrosomes are not stained whereas sperm with acrosomes, are (see diagram). On the contrary, in the direct functional assay, PSA reaches the acrosomal matrix of sperm undergoing exocytosis *without fixation*: only these sperm stain. This is because PSA enters the acrosomes through the fusion pores and remains attached to the matrix (see diagram) (Sanchez-Cardenas et al., 2014; Pelletan et al., 2015; Sosa et al., 2015). Thus, the read outs of both protocols are complementary. Sperm loaded with anti-α-synuclein antibodies and challenged with CaCl_2_ evinced fluorescent acrosomes under both staining methods: cells incorporated the lectin through the fusion pores (direct protocol, Figure 4B); yet, they retained their acrosomal matrix (indirect staining, Figure 4A). Taken together these results support our hypothesis that the exocytotic cascade advanced to a post-fusion—and before acrosomal dispersion—stage. Similar results are obtained when permeabilized sperm are incubated with the chimaeric protein Rab3A-22A before challenging with CaCl_2_ (Quevedo et al., 2016).

We resorted to transmission electron microscopy to gain further insights into the exocytotic stage halted by the anti-α-synuclein antibodies. Micrograph labeled “intact” in Figure 4C shows the typical aspect of the heads of a resting cell, where the acrosome is planar. This pattern is the most abundant in unperturbed cells (Figure 4D, I). The cell photographed in the panel “fully exocytosed” has completed the AE. As expected, this stage was predominantly observed in samples challenged with calcium (Figure 4D, IV). Panel “swollen and waving” illustrates a cell that underwent acrosomal swelling/deformation in response to external calcium, but it could not complete the AE. This pattern is seldom observed in cells undergoing exocytosis spontaneously and those challenged with calcium, because swelling is transient (Figure 4D, II) and (Sosa et al., 2015). Interestingly, this was the predominant pattern in cells preloaded with anti-α-synuclein antibodies before adding CaCl_2_ (Figure 4D, black bars). This response resembled that observed when using recombinant Rab3A-22A instead of anti-α-synuclein antibodies (Quevedo et al., 2016). Few cells completed the AE because the antibodies prevented it. We visualized diffuse, localized and electron dense material adhered to the surface of a subpopulation of cells with swollen and waving acrosomes, exclusively upon treatment with anti-α-synuclein antibodies and calcium (Figure 4C, pattern “swollen, waving and diffuse (III)”, arrows, quantified in Figure 4D, III).

**FIGURE 5:**
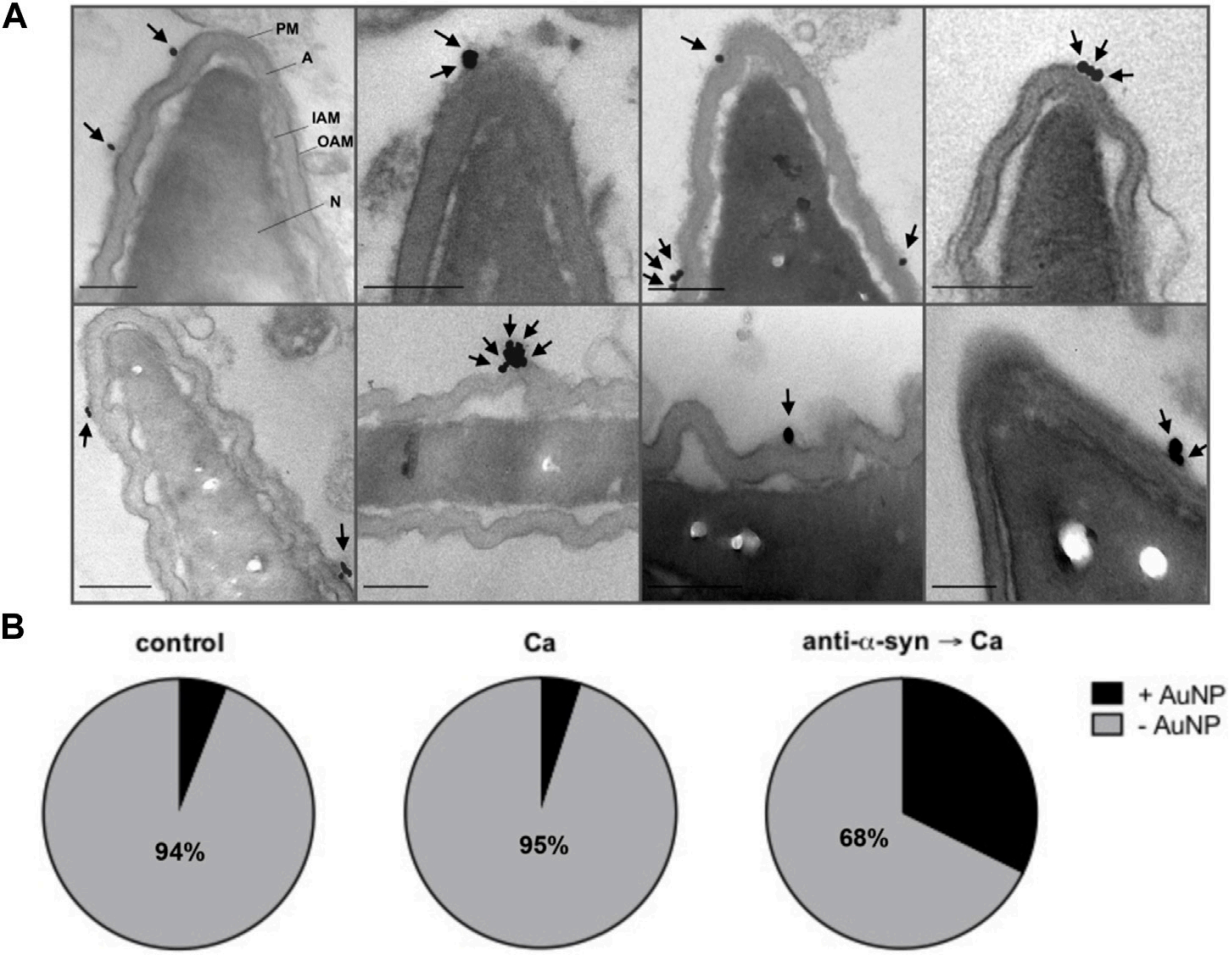
PSA-gold binds the electron dense material that diffuses out of sperm. Spermatozoa were incubated for 15 min at 37°C with culture medium or 15 nM anti-α-synuclein antibodies (α-syn) followed by 20 μg/mL PSA-gold nanoparticles (AuNP) and a 15 min at 37°C incubation. When indicated, 0.5 mM CaCl_2_ (Ca) was added and incubations continued for 30 min at 37°C. **(A)** Gallery showing gold nanoparticles (arrows) bound to the surface in the acrosomal region of sperm heads with swollen and waving acrosomes. Bars = 200 nm. **(B)** Quantification: grey area: cells without gold nanoparticles (-AuNP, numbers indicate the percentage of cells in this category); black area: cells with gold nanoparticles (+AuNP). “Control” 102 cells scored, “Ca”, 101 cells scored, “anti-α-syn→Ca”, 102 cells scored. Statistical analysis was performed using GraphPad Prism. Chi-square test was applied for proportions. *p*-value < 0.0001 were considered statistically significant.

Considering that the anti-α-synuclein antibodies stabilized open—but not expanded—fusion pores (Figure 4B), we hypothesized that the electron dense material characteristic of pattern III might correspond to acrosomal components that diffuse out of the cells through those pores. To test this premise, we adapted for electron microscopy experiments similar to those summarized in Figure 4B, only that PSA coupled to 20 nm colloidal gold particles substituted for FITC-PSA. The membranes of intact sperm (pattern I in Figure 4C) showed absence of gold nanoparticles (images not shown but included in quantifications to compose Figure 5B), which indicates that they were not ruptured by SLO-permeabilization or processing for transmission electron microscopy. Likewise, PSA-gold did not bind sperm that had completed the AE (pattern IV in Figure 4C; images not shown but included in quantifications to compose Figure 5B), which suggests that no acrosomal matrix remained associated to the inner acrosomal membrane. Interestingly, one or more gold particles were consistently found adjacent to the surface of cells exhibiting swollen and waving acrosomes (patterns II and III in Figure 4C, undistinguishable here). A gallery of typical images illustrating this subpopulation of sperm is shown in Figure 5A, with arrows pointing to gold nanoparticles. Quantification of hundreds of electron micrographs showed that the proportion of gold-labelled cells (+AuNP) was low in sperm that underwent no treatment (Figure 5B, control) or were exposed to the AE inducer (Figure 5B, Ca). This is consistent with the observations that bins II and III were the least abundant of the histogram shown in Figure 4C. In contrast, gold nanoparticles decorated the extracellular face of the acrosomal region in a high percentage of cells that had initiated the AE but could not complete it because the anti-α-synuclein prevented it (Figure 5B, anti-α-syn → Ca). This is consistent with the distribution shown in Figure 4C, where bins II and III were the most abundant under these experimental conditions. Because colloidal gold was coupled to a probe that exclusively binds the acrosomal matrix, these results support our hypothesis that the acrosomal contents targeted by PSA had diffused out of the sperm (diffuse material, arrows in pattern III, Figure 4C). Similar patterns of detection of extracellular acrosomal contents attributed to efflux through fusion pores have been described with anti-acrosin antibodies (Tesarik et al., 1988) and PSA coupled to paramagnetic beads (Kohn et al., 1996). Colloidal gold present in the medium bathing the cells was seldom observed inside acrosomes, which reflects its inability to traverse the pores opened between the plasma and acrosomal membranes. Soluble PSA permeated through fusion pores and reached the acrosomal membrane (Figure 4B) but that bound to gold nanoparticles did not; these observations suggest that the latter was excluded because of size. The pores were large enough to allow efflux of acrosomal material but too small to allow influx of 20 nm gold nanoparticles. Thus, we conclude that the fusion pores stabilized by anti-α-synuclein antibodies were smaller than 20 nm, the size of the gold nanoparticles used in this study.

### α-Synuclein is required for fusion pore dilation

We have shown that the inhibition of the AE by anti-α-synuclein antibodies is a post-fusion event because they freeze sperm SNAREs in toxin-resistant, *cis* complexes (Figure 3) and allow the influx of an acrosomal probe (Figure 4B) as well as the efflux of the acrosomal matrix (Figure 5). Is α-synuclein required after bilayer fusion to dilate the fusion pores? We tested this hypothesis with computational and experimental approaches. We have recently implemented into PLUMED the reaction coordinate ξ_e_ to study the thermodynamics of the expansion of the fusion pore using restrained molecular dynamics (Di Bartolo et al., 2022). ξ_e_ is a dimensionless parameter approximately proportional to the radius (R, in nm) of a toroid-shaped fusion pore (see Eq. 1) [for mathematical details on ξ_e_ see Masone and collaborators (Di Bartolo et al., 2022)]

$$R≃0.75\xi_{e}+0.2$$ (1)

**FIGURE 6:**
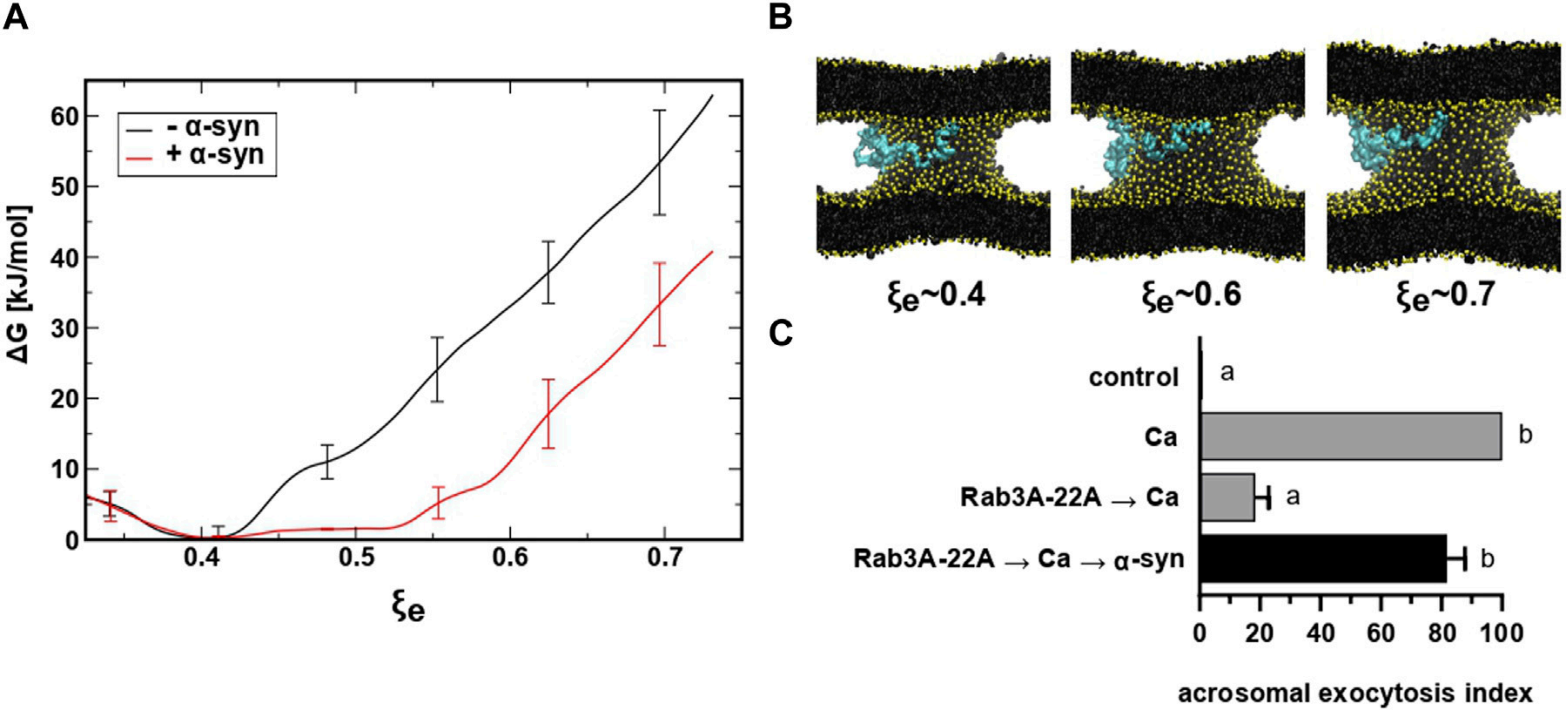
restrained molecular dynamics simulations reveal that α-synuclein favors the expansion of the fusion pore. **(A)** Free energy profiles for fusion pore expansion with (red line) and without (black line) full-length α-synuclein. **(B)** Molecular dynamics snapshots for different stages of the expanded fusion pore. Lipids are colored in black with phosphate groups in yellow and α-synuclein in cyan. For clarity, water molecules are not shown. **(C)** SLO-permeabilized sperm were incubated with 300 nM Rab3A-22A, 0.5 mM CaCl_2_, and 50 nM α-synuclein (10 min at 37°C after each addition, black bar). Gray bars represent controls. The AE was measured as indicated in the legend to Figure 2A.

To investigate the influence of α-synuclein, we expanded a fusion pore nucleated between two lipid bilayers using molecular dynamics simulations and the collective variable ξ_e_. We inserted a Martini model of α-synuclein between the bilayers (in the cytosolic space) generated from the human micelle-bound α-synuclein (PDB ID: 1xq8) (Ulmer et al., 2005). Figure 6A shows the Gibbs free energy needed to expand a fusion pore with (red) and without (black) α-synuclein in system. A significant reduction (∼ 20 kJ/mol) of the free energy occurred as the fusion pore expanded from ξ_e_ ∼ 0.4 (R ≃ 0.5 nm) to ξ_e_ ∼ 0.75 (R ≃ 0.75 nm) in the presence of the protein. Snapshots of the expanding pore at three different values of ξ_e_ are shown in panel B. Notice that the protein, initially situated in the “cytoplasm” (between the bilayers), freely adsorbed on the phospholipid surface of the pore. This location is consistent with experimental data that show strong binding of α-synuclein to highly curved lipid membranes (Middleton and Rhoades, 2010; Caparotta et al., 2020a). These findings suggest that α-synuclein facilitates the expansion of fusion pores that connect two pure lipid membranes.

When introduced into SLO-permeabilized human sperm, the chimeric protein Rab3A-22A halts the AE elicited by calcium at a stage after membrane fusion/pore opening but before pore expansion/membrane vesiculation (Quevedo et al., 2016). Addition of recombinant α-synuclein after Rab3A-22A and calcium rescued the exocytotic block imposed by the chimaera (Figure 6C). Taken together, our results suggest that α-synuclein is required at a post-fusion stage during sperm exocytosis and that its unavailability inhibits the AE because pores do not expand.

## Discussion

Exocytosis involves fusion of cargo-loaded vesicles and granules with the plasma membrane. Despite similar molecular machineries, exocytosis proceeds at different speeds, being much faster in synaptic vesicles (∼40 nm diameter, high curvature) than in chromaffin and insulin granules (∼100–300 nm diameter, low curvature) (Kreutzberger et al., 2019). The fusion pore that opens between the fusing membranes and connects the granule’s lumen with the extracellular space can evolve in various ways: it can dilate—slowly or rapidly—to allow full cargo release (full fusion), it can reseal (kiss and run) or open and close repeatedly (flickering) (Karatekin, 2018; Mion et al., 2022). Among other factors, pore dynamics, vesicle size and curvature of the membrane determine the kinetics of cargo release. The acrosome is a very large, electron dense granule that surrounds the elongated nucleus in the apical domain of the sperm head. The head of the human sperm is oval, 3.7–4.7 μm long and 2.5–3.2 μm wide, with the acrosomal domain comprising 40%–70% of the head (Bellastella et al., 2010). Owing to its large size, exocytosis of the acrosome is very slow (minutes instead of seconds or milliseconds) (Harper et al., 2008; Sosa et al., 2015; Romarowski et al., 2016). Rather than being spherical like most vesicles and granules, it resembles a horseshoe in longitudinal sections of resting sperm observed by transmission electron microscopy. The anterior acrosome, or acrosomal cap, is involved in AE, whereas the posterior acrosome, or equatorial segment, is involved in fusion with the oolema. The acrosomal membrane of resting cells exhibits low curvature, except for the anterior tip, the region where fusion with the plasma membrane begins in human sperm (Yudin et al., 1988; Harper et al., 2008). Once the AE has initiated, the acrosome swells and its outer membrane waves profusely, acquiring high curvature. Swelling, fusion and vesiculation do not extend to the equatorial segment, which remains intact after AE, with plasma, inner and outer acrosomal membranes close to one another in a parallel, linear array (Yudin et al., 1988). As stated before, the fate of the fusion pores that open during the AE does not fit into full fusion, kiss and run or flickering modes. Despite these differences, exocytosis of the acrosome relies on the same molecular machinery as that in endocrine and neuroendocrine cells and also depends on the opening and dilation of fusion pores between the acrosomal and plasma membranes.

Modulation of fusion pore opening and dilation during exocytosis has been attributed to various members of the fusion machinery (reviewed in (Karatekin, 2018; Chowdhury and Zorec, 2020; Risselada and Mayer, 2020)), Epac2 (Gucek et al., 2019) and α-synuclein. A study conducted in rat hippocampal primary neurons and mouse adrenal chromaffin cells shows that both, overexpressed and endogenous α-synucleins accelerate the dilation of the fusion pores, whereas loss of all three synuclein isoforms increases the likelihood of pore closure events without affecting the time to closure. Fusion pore dilation would not be expected to influence the release of classical transmitters, such as glutamate, that escape rapidly even through small pores. In contrast, it would affect the release of monoamines and peptides that dissociate slowly from a luminal matrix (Logan et al., 2017). Likewise, in an *in vitro* system that uses TIRF microscopy to study the fusion between single vesicles containing synaptobrevin-2 and flat artificial membranes containing syntaxin1a and SNAP-25, α-synuclein promotes the probability of opening, duration and expansion of large (6 nm) fusion pores (Khounlo et al., 2021). Lastly, overexpression of α-synuclein quickens the post-fusion discharge of brain-derived neurotrophic factor in bovine chromaffin cells detected by videomicroscopy in live cells (Abbineni et al., 2019). Although these observations are consistent with an increase in fusion pore expansion, the authors of this report failed to capture such expansion by TIRF microscopy.

α-Synuclein adopts a bent helix structure when bound to highly curved micelles but adopts an elongated helix structure when bound to membranes with low curvature (Trexler and Rhoades, 2009). It has been hypothesized that at the highly curved membranes surrounding the fusion pore, α-synuclein adopts a bent α-helix structure that binds to the vesicle on one side and to the plasma membrane on the other. Its transition to an extended state would drive pore dilation (Runwal and Edwards, 2021). The authors of this very insightful review put forth an alternative mechanism by which α-synuclein may regulate SNARE complex dynamics after fusion (Runwal and Edwards, 2021). Given the high curvature of the outer membrane in swollen acrosomes (Figures 4C, 5) and the resistance of synaptobrevin to toxin cleavage (Figure 3) in sperm treated with anti-α-synuclein and calcium, both hypotheses would be worth exploring.

Despite its small size, α-synuclein failed to diffuse out of SLO-permeabilized human sperm (Figure 1C), perhaps reflecting its interaction with sperm membranes. Alternatively, α-synuclein may engage in large molecular weight complexes unable to exit the cell through the toxin-generated pores. No loss of protein by diffusion accounted for the access of the anti-α-synuclein antibodies to their intracellular target. By obstructing the function of sperm α-synuclein, the antibodies inhibited the AE because they impaired a post-docking (Figure 2B), post-fusion pore opening (Figures 4A, B) step of the pathway. It is licit to speculate that the electron dense material appreciated in the “swollen, waving and diffuse” classification in Figure 4 may correspond, at least partially, to the acrosomal contents recognized by the gold-coupled lectin (Figure 5). Taken together, these findings strongly suggest that treatment with anti-α-synuclein antibodies stabilizes fusion pores during the calcium-induced AE. It was through these pores that FITC-PSA entered (Figure 4B) and electron dense material diffused out of (Figure 5) the acrosomal granule. These findings are reminiscent of those attained when Rab3A-22A substituted for anti-α-synuclein antibodies (Quevedo et al., 2016). Addition of recombinant α-synuclein rescued the block imposed by the antibodies (Figure 3A). One possible explanation for these findings is that the recombinant protein (which did not influence the percentage of AE *per se* in our end-point assay, see Supplementary Figure S2) added at the end of the incubation displaced the antibody from sperm synuclein and allowed the exocytotic cascade to resume. Exogenous α-synuclein also rescued the block imposed by Rab3A-22A (Figure 6C), which supports the more attractive notion that the protein directly or indirectly expanded the pores. Results from computational analysis also agree with this view (Figures 6A, B).

The novel findings reported here are: i) α-synuclein regulates a post-fusion stage of the AE to dilate the pores; ii) when endogenous α-synuclein is unavailable, SNARE proteins are engaged in post-fusion *cis* complexes; iii) when endogenous α-synuclein is unavailable, electron dense contents diffuse out of the acrosome through open, stable fusion pores. As happens in other cells, sperm fusion pores are subjected to post-fusion regulation.

Misfolded α-synuclein species can spread between cells in a prion-like manner and seed the aggregation of endogenous protein in recipient cells. This cell-to-cell transmission and propagation of misfolded α-synuclein mirrors the spread of human neurodegenerative diseases such as Parkinson’s (Bras et al., 2020; Oliveira et al., 2021). Many pathogenic mutations have been discovered in the α-synuclein-encoding gene *SNCA* in cases of familial Parkinson’s disease. *SNCA* overexpression and its mutants were introduced in animal models of neurodegeneration [reviewed in (Oliveira et al., 2021) (Runwal and Edwards, 2021; Gasser, 2023]. Neither aggregation of synuclein in the male germline nor the fertility of these animals have been reported to date. Single α-synuclein (Abeliovich et al., 2000), double αγ-synuclein (Papachroni et al., 2005) and triple αβγ-synuclein (Greten-Harrison et al., 2010) null mice are viable and fertile. We would have expected reduced male fertility in these cases. However, overt reproductive phenotypes are rarely observed in mating experiments even when egg- or sperm-specific proteins with well established roles in fertility are knocked out. In contrast, problems are readily detected when gametes of deficient animals are tested in experiments designed to assess their fertility, such as IVF, something that has not been done for synucleins. This is an interesting avenue to pursue in the future.

## Data Availability

The original contributions presented in the study are included in the article/Supplementary Material, further inquiries can be directed to the corresponding author.

## Abbreviations

AE, acrosome exocytosis; BoNT/B, botulinum toxin B; BSA, bovine serum albumin; FITC, fluorescein isothiocyanate; HTF, human tubal fluid; IPTG, isopropyl-β-D-thio-galactoside; NP-EGTA-AM, O-nitrophenyl EGTA-acetoxymethyl ester; PBS, phosphate buffer saline; PSA, *Pisum sativum* agglutinin; SDS-PAGE, sodium dodecyl sulfate-polyacrylamide gel electrophoresis; PVP, polyvinylpyrrolidone; SLO, streptolysin O; SNARE, SNAP receptor; TeTx, tetanus toxin.
