# Supplementary figures and images for: α-Synuclein is required for sperm exocytosis at a post-fusion stage
Source: Front Cell Dev Biol. 2023 May 23;11:1125988. doi: 10.3389/fcell.2023.1125988 (PMC10242118; doi:10.3389/fcell.2023.1125988)

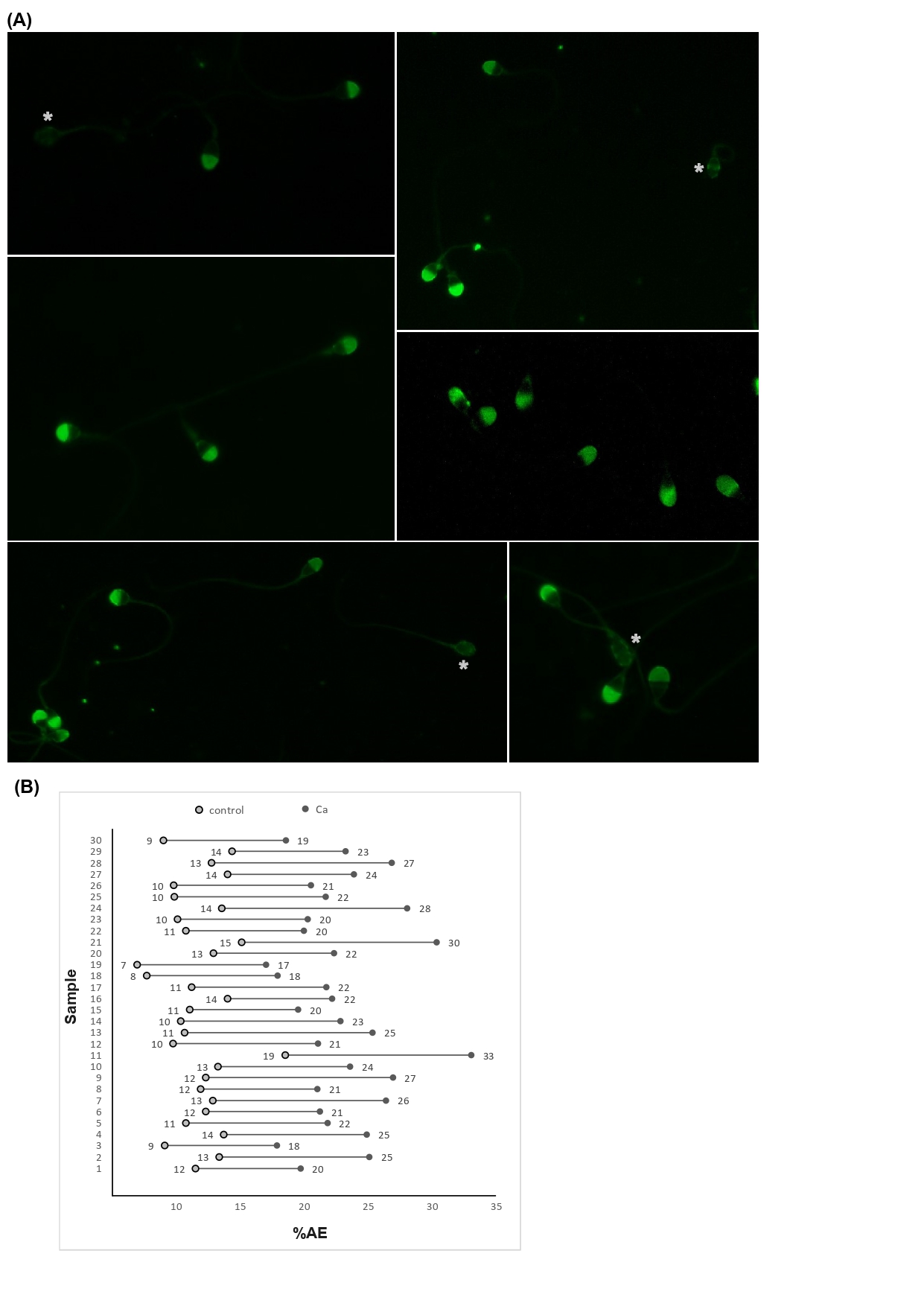

Supplement: Supplementary file 1 [file Image1.JPEG]

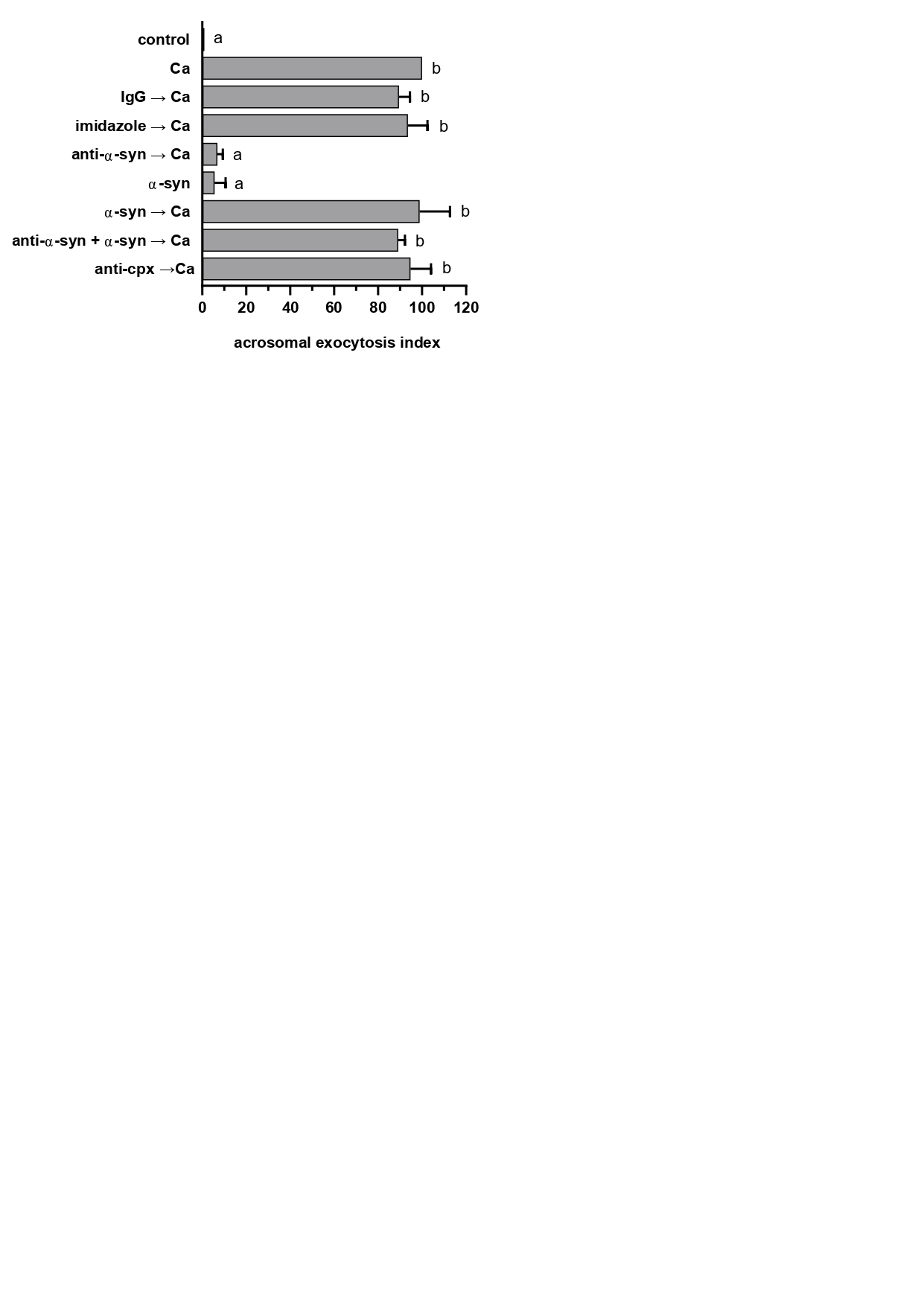

Supplement: Supplementary file 2 [file Image2.JPEG]
