# Supplementary material for: α-Synuclein is required for sperm exocytosis at a post-fusion stage
Source: Front Cell Dev Biol. 2023 May 23;11:1125988. doi: 10.3389/fcell.2023.1125988 (PMC10242118; doi:10.3389/fcell.2023.1125988)
Supplement: Supplementary file 3 [file DataSheet1.DOCX]

Supplementary information

Supplementary Materials and methods

Molecular dynamics simulations followed a semi-isotropic NPT ensemble with a time step of 20fs. All systems were initially prepared using CHARMM-GUI web-based interface (Jo et al., 2008). The temperature was set at T=303.15K (Wu et al., 2014) and controlled by a V-rescale thermostat (Bussi et al., 2007) using a coupling constant of 1ps. The pressure was set at 1.0bar with a compressibility of to 3x10^−4^ bar^−1^, using the Parrinello-Rahman barostat (Parrinello and Rahman, 1981) with a 12ps time constant. Neighbor search used the Verlet cut-off scheme with a buffer tolerance of 0.005kJ/mol/ps and a 20 step update-frequency of the neighbor list. Periodic Boundary Conditions were used in all directions. Coulomb interactions used the reaction field method with a cut-off of 1.1nm. Van der Waals interactions used the cut-off scheme set to 1.1 nm.

Supplementary Figure legends

**Legend to Figure S1: (A)** FITC-PSA staining of SLO-permeabilized sperm. Gallery showing acrosomal staining of human sperm that have (asterisks) or have not (the rest) undergone AE. Shown are six representative fields.

**(B)** Dumbbell plot of the percentages of AE from 30 representative samples used for indirect AE assay experiments. The y-axis shows the sample number and the x-axis shows percentages of AE, with the dumbbell plot connecting the values from the basal control (open circles) to stimulated with calcium (positive control, closed circles) within a sample.

**Legend to Figure S2: specificity controls.** SLO permeabilized human sperm were treated for 15 min at 37 °C in the presence of 7.5 nM nonimmune rabbit IgG, 27.8 mM imidazole (the concentration present in purified 20 nM α-synuclein), 7.5 nM anti-α-synuclein antibodies-pretreated (anti-α-synuclein + α-synuclein) or not with 20 nM α-synuclein, 7.5 nM anti-complexin I/II antibodies (anti-cpx) or 20 nM α-synuclein before challenging with 0.5 mM CaCl_2_ for an additional 15 min. Treatment with 20 nM α-synuclein alone was also tested. Cells were fixed and acrosomal exocytosis was evaluated by FITC-PSA binding (indirect method). The data represent the mean ± SEM of at least three independent experiments. Different letters indicate statistical significance (p<0.001).

References

Bussi, G., D.Donadio, and M.Parrinello. 2007. Canonical sampling through velocity rescaling. *J. Chem. Phys.* 126:014101.

Jo, S., T.Kim, V.G.Iyer, and W.Im. 2008. CHARMM-GUI: a web-based graphical user interface for CHARMM. *J. Comput. Chem.* 29:1859-1865.

Parrinello, M., and M.S.Rahman. Polymorphic transitions in single crystals: A new molecular dynamics method. Journal of Applied Physics 52, 7182-7190. 1981.

Ref Type: Generic

Wu, E.L., X.Cheng, S.Jo, H.Rui, K.C.Song, E.M.Davila-Contreras, Y.Qi, J.Lee, V.Monje-Galvan, R.M.Venable, J.B.Klauda, and W.Im. 2014. CHARMM-GUI Membrane Builder toward realistic biological membrane simulations. *J. Comput. Chem.* 35:1997-2004.
